# Editorial: Neurotrauma and repair: from peripheral to central nerve injury

**DOI:** 10.3389/fnmol.2023.1338869

**Published:** 2023-12-01

**Authors:** Alexander Younsi

**Affiliations:** Department of Neurosurgery, Heidelberg University Hospital, Heidelberg, Germany

**Keywords:** neurotrauma, neuroregeneration, spinal cord injury, traumatic brain injury, peripheral nerve injury

Neurotrauma, encompassing traumatic brain injury (TBI), peripheral nerve injury (PNI), and spinal cord injury (SCI), continues to be a major global public health concern. The complex pathophysiological mechanisms and lifelong neurological deficits associated with neurotrauma emphasize the urgent need for effective clinical interventions. The Research Topic “*Neurotrauma and Repair: from Peripheral to Central Nerve Injury*” brought together a diverse group of 58 authors, fostering interdisciplinary collaboration and offering new insights into the multifaceted landscape of neurotrauma research.

This collaborative effort yielded a body of work that has garnered significant attention within the scientific community, underscoring the importance of the research contributions and the demand for advances in neurotrauma understanding and treatment.

“*Effects of a Neurokinin-1 Receptor Antagonist in the Acute Phase after Thoracic Spinal Cord Injury in a Rat Model*” delves into the potential therapeutic effects of neurokinin-1 receptor antagonists on neuroinflammation and edema following SCI (Zheng et al.). While the study suggests limited impact on reducing edema, it demonstrates a notable reduction in T-lymphocyte invasion and apoptotic cell numbers with the treatment. Additionally, it suggests a trend toward reduced fibrinogen leakage, endothelial and microglial activation, chondroitin sulfate glycosaminoglycan deposition, and astrogliosis. Although general locomotion recovery remained modest, CatWalk gait analysis revealed early improvements in several parameters. This research showcases the intricate and multifaceted nature of SCI and the potential of neurokinin-1 receptor antagonists in mitigating some aspects of neurogenic inflammation.

In “*Muscle-Derived Stem Cell Exosomes with Overexpressed miR-214 Promote the Regeneration and Repair of Rat Sciatic Nerve after Crush Injury*,” researchers explore the regenerative potential of muscle-derived stem cell exosomes enriched with miR-214 (Zeng et al.). The study demonstrates the ability of these exosomes to promote the proliferation and migration of Schwann cells, enhance the expression of neurotrophic factors, and facilitate axon extension in dorsal root ganglion neurons. By targeting PTEN and activating the JAK2/STAT3 pathway, these exosomes hold promise for promoting nerve regeneration in the context of sciatic nerve crush injuries. This research not only contributes to our understanding of neural repair mechanisms but also offers a potential avenue for therapeutic intervention.

“*Partially Brain Effects of Injection of Human Umbilical Cord Mesenchymal Stem Cells at Injury Sites in a Mouse Model of Thoracic Spinal Cord Contusion*” takes an intriguing angle, investigating the consequences of SCI on the brain (Hu et al.). This study explores the potential therapeutic mechanisms of human umbilical cord mesenchymal stem cell injection at the injury site. It highlights the intricate interactions between the spinal cord and the brain, underscoring the interconnectedness of neural systems. This research raises important questions about the broader neurological effects of spinal cord injuries and offers insights into potential therapeutic interventions that could extend beyond the immediate injury site.

“*A Closed-Body Preclinical Model to Investigate Blast-Induced Spinal Cord Injury*” introduces an innovative preclinical model for studying blast-induced spinal cord injuries (Norris et al.). These injuries are particularly relevant in military contexts, where exposure to blast overpressure can result in significant spinal trauma. This research enhances our understanding of blast dynamics and their effects on the spinal cord, providing valuable insights into the complexities of these injuries. By investigating markers of traumatic axonal injury and neuroinflammation, this study sets the stage for future research into minimally invasive treatment approaches.

In “*Huntingtin-Associated Protein 1 Ameliorates Neurological Function Rehabilitation by Facilitating Neurite Elongation*,” researchers explore the role of Huntingtin-associated protein 1 (HAP1) in promoting neurological function recovery (Miao et al.). By activating the TrkA-MAPK signaling pathway, HAP1 demonstrates its potential to enhance neurite elongation and improve outcomes in SCI. This study not only advances our understanding of the molecular mechanisms underlying neural repair but also suggests a promising avenue for therapeutic intervention.

“*Differential Effects on TDP-43, Piezo-2, Tight-Junction Proteins in Various Brain Regions Following Repetitive Low-Intensity Blast Overpressure*” delves into the often-overlooked issue of mild traumatic brain injuries caused by repetitive low-intensity blast overpressure (Heyburn et al.). This research highlights the varied effects of such exposures on critical proteins in different brain regions, underscoring the structural and anatomical heterogeneity of the brain. The findings emphasize the need for a comprehensive understanding of neurotrauma's diverse impacts, especially in military and combat-related contexts.

In the review article “*A Cutting-Edge Strategy for Spinal Cord Injury Treatment: Resident Cellular Transdifferentiation*,” the authors explore a cutting-edge strategy for SCI treatment—resident cellular transdifferentiation (Fang et al.). This innovative approach involves reprogramming mature somatic cells into functional neurons, offering new hope for post-traumatic spinal cord repair and functional improvement. While the mechanisms and induced neuronal subtypes are not yet fully understood, this review provides a comprehensive analysis of the current state of research in this exciting field.

These contributions collectively advance our understanding of neurotrauma and offer new insights into potential pathways for treatment. While challenges remain, this collaborative effort signifies progress in the field, underscoring our shared commitment to alleviating the burden of neurotrauma for individuals worldwide. As we reflect on these achievements, we acknowledge the road ahead, filled with challenges and opportunities. Neurotrauma research continues to evolve, driven by a global community of researchers dedicated to translating insights into tangible treatments. Together, we embark on a journey that promises to bring healing and recovery to those affected by neurotrauma, one breakthrough at a time.

## Author contributions

AY: Writing—original draft.

